# The allosteric AKT inhibitor MK2206 shows a synergistic interaction with chemotherapy and radiotherapy in glioblastoma spheroid cultures

**DOI:** 10.1186/s12885-017-3193-9

**Published:** 2017-03-21

**Authors:** Ravi S. Narayan, Carlos A. Fedrigo, Eelke Brands, Rogier Dik, Lukas J.A. Stalpers, Brigitta G. Baumert, Ben J. Slotman, Bart A. Westerman, Godefridus J. Peters, Peter Sminia

**Affiliations:** 1 Department of Radiation Oncology, VU University Medical Center/Cancer Center Amsterdam, P.O. Box 7057, Amsterdam, 1007 MB The Netherlands; 2 Department of Radiation Oncology, Academic Medical Center, Amsterdam, The Netherlands; 3 Clinical Cooperation Unit Neurooncology, MediClin Robert Janker Klinik & University of Bonn Medical Center, Bonn, Germany; 4 Department of Radiation Oncology, Maastro Clinic, Maastricht, The Netherlands; 5 Department of Neurosurgery, Neuro Oncology Research Group, VU University Medical Center, Amsterdam, The Netherlands; 6 Department of Medical Oncology, VU University Medical Center, Amsterdam, The Netherlands

**Keywords:** Glioma, Radiosensitization, Akt, Spheroid cultures, MK2206, Synergy

## Abstract

**Background:**

Glioblastoma multiforme (GBM) is the most common, invasive and deadly primary type of malignant brain tumor. The Phosphatidylinositol-3-Kinase/AKT (PI3K/AKT) pathway is highly active in GBM and has been associated with increased survival and resistance to therapy. The aim of this study is to investigate the effects of AKT inhibition in combination with the current standard of care which consists of irradiation and temozolomide (TMZ) on human malignant glioma cells growing adherent and as multicellular spheroids in vitro.

**Methods:**

The effects of the allosteric inhibitor MK2206 combined with irradiation and TMZ were assessed on glioma cells growing adherent and as multicellular 3D spheroids. The interaction was studied on proliferation, clonogenic cell survival, cell invasion, −migration and on expression of key proteins in the PI3K-AKT pathway by western blot.

**Results:**

A differential effect was found at low- (1 μM) and high dose (10 μM) MK2206. At 1 μM, the inhibitor reduced phosphorylation of Thr308 and Ser473 residues of AKT in both adherent cells and spheroids. Low dose MK2206 delayed spheroid growth and sensitized spheroids to both irradiation and TMZ in a synergistic way (Combination index <0.35). In contrast, neither low nor high dose MK2206 did enhance therapy sensitivity in adherent growing cells. Effective inhibition of invasion and migration was observed only at higher doses of MK2206 (>5 μM).

**Conclusions:**

The data show that a 3D spheroid model show different sensitivity to irradiation when combined with AKT inhibition. Thereby we show that MK2206 has potential synergistic efficacy to the current standard of care for glioma patients.

**Electronic supplementary material:**

The online version of this article (doi:10.1186/s12885-017-3193-9) contains supplementary material, which is available to authorized users.

## Background

Glioblastoma multiforme (GBM) is the most common and aggressive primary brain tumor in adults, with an overall incidence rate of approximately 3 per 100,000 persons per year [1]. The unique characteristics of GBM, such as high mitotic capacity, microvascular proliferation, pseudopallisading necrosis and infiltrative growth, confer a poor prognosis, with a median overall survival of approximately 15 months after diagnosis [2]. Postoperative radiotherapy (RT) with concomitant temozolomide (TMZ) has become the standard procedure in the treatment of patients with newly diagnosed GBM, based on the results of a large European-Canadian phase III trial [3]. Despite these encouraging results, the majority of patients still succumb from locally recurrent disease, which is due to the diffuse infiltrative growth characteristics of this tumor type and high level of resistance to radiotherapy and chemotherapy [4]. The treatment response and prognosis are related to several (epi)genetic characteristics of glioma like methylation status of O6-methylguanine–DNA methyltransferase (MGMT) and genetic events in GBM core pathways including the phosphatidylinositide 3-kinase (PI3K) pathway [5]. PI3K is a central upstream node related to cell survival and cell proliferation [6]. Its primary downstream effector protein AKT plays a pivotal role in the pathway activation via phosphorylation of AKT on two critical residues, Thr308 (through PI3K) and Ser473 (mediated predominantly via mTORC2) [6, 7]. AKT exists in three isoforms, AKT1, −2 and −3, of which AKT2 and −3 are found to be important in glioma cells [7, 8]. Experimental data has indicated that phosphorylated AKT is required for proper DNA-damage response (DDR) during Non-Homologous end-joining (NHEJ) by binding to DNA-PKcs and promoting its auto-phosphorylation [9, 10]. Pharmacological inhibition of AKT has therefore also been found to sensitize cancer cells to DNA damaging agents and radiotherapy [11, 12]. In recent years many specific PI3K/AKT/mTOR pathway targeted agents have become available for preclinical studies and clinical evaluation [13]. MK2206 is an oral allosteric AKT inhibitor which can inhibit all isoforms of AKT [14]. Early clinical feasibility studies already demonstrated that MK2206 monotherapy is well tolerated in patients [15]. Emerging data show MK2206 to enhance the activity of chemotherapeutic agents in various types of cancers both pre-clinical [14, 16–21] and in patients [15, 22]. Data on MK2206 additional to the current standard GBM therapy are however not available. In the present study, we investigated the effect of MK2206 alone and its ability to synergize with radiation and TMZ to inhibit glioma growth, invasion and migration using monolayer human glioma cells and multicellular glioma spheroids.

## Methods

### Monolayer and spheroid/organoid cell culture

Experiments were performed using the established glioma cell lines U87MG (ATCC-HTB-14) and U251 (cell line was kindly provided by Dr. C.H. Langevel, Dept. Neurology, VU University Medical Center, Amsterdam, The Netherlands) and on two primary cell lines VU28 and VU122 (derived directly from surgical specimens from the VU University Medical Center). Cells were cultured at 37 °C in Dulbecco’s modified Eagle’s medium (D-MEM; Gibco BRL, UK) containing 10% fetal calf serum, 100 IU ml – 1 penicillin and 100 IU ml – 1 streptomycin, in at 5% CO2-humidified atmosphere. The AKT-inhibitor MK-2206 (Selleck Chemicals®, Houston, Texas, USA) was dissolved to a 10 mM stock solution in DMSO and stored at −20 °C. The alkylating agent temozolomide (Schering-Plough®, Utrecht, The Netherlands) was freshly dissolved at 100 mM in DMSO before each treatment. Cells and spheroids were irradiated at room temperature radiation from a Cobalt-60 source at a dose rate of 516 Gy/h (Gammacell 220®; Atomic Energy of Canada, Mississauga, Ontario, Canada).

### Cell proliferation

U87MG Cells were plated at a density of 2000 cells/well in a 96-well plate 24 h prior to drug treatment. Subsequently, cells were exposed to a serial dilution of MK2206 for 72 h in sextuple. Cell viability was determined using Cell-titer Glo 3D (Promega), which dissociates the spheroids. Relative light units (RLU) were measured using the BioTek Synergy HT Microplate Reader RLUs were normalized against the untreated controls.

### Western blot

Expression of total AKT (Cell Signaling #9272, Boston, USA, 60 kDa), phospho-AKT Ser473 (Cell Signaling #9271, 60 kDa), phospho-AKT Thr308 (Cell Signaling #9275, 60 kDa), phospho-H2A.X (#9718) with loading control total-S6 (#2217) proteins were evaluated by western blot. Cells were either treated with 1 μM MK-2206 1 h before being irradiated, and collected at indicated time points for analysis.

### Migration assay

Cells were plated at high density of 30.000 cells/well in 96-wells plates. A day later wells were uniformly scratched using a guided 96-well pin tool (Peira, Turnhout, Belgium) to create wounds of approximately 300 μm wide. Wells were washed with PBS and growth medium was added with MK2206. Images were automatically captured on a Leica DMI3000 microscope (Leica, Rijswijk, The Netherlands) using Universal Grab 6.3 software (DCILabs, Keerbergen, Belgium). Scratch sizes were determined using Scratch Assay 6.2 (DCILabs), and absolute wound closure (μm^2^) was expressed as a percentage of control wells.

### Invasion assay

Cellular invasion was evaluated using Boyden chamber assay. In short, 2 × 10^5^ cells were seeded into each insert of a 24-well plate (Falcon #353504, Fisher Scientific, USA) containing serum-free medium in the upper compartment and complete medium in the lower compartment, separated by a matrigel (10%) membrane in D-MEM. MK-2206 (1–10 μM) was added to both compartments. After 16 h of cell seeding the invasive capacity was assessed using a fluorescent microscope to count the number of cells that crossed the membrane. Fluorescence was achieved with addition of 5 μM Calcein-AM to the lower compartment in the last half hour of the experiment. For the combination with irradiation, exponential growing cells were exposed to 1 μM of MK-2206 for 1 h or 24 h, followed by 4 Gy irradiation and incubation at 37 °C for one hour and then transferred to inserts.

### Spheroid growth and migration

U87MG tumor spheroids were prepared from monolayer cells which were trypsinised and seeded at a density of 5 × 10^6^ cells/well in 6-well ultra-low attachment plate (Corning #3471, Boston, USA) containing complete DMEM. After 2 days, round spheroids were formed and those with 150–250 μm diameter were collected with a micropipette in an inverted microscope, transferred and cultured individually again with complete DMEM in 24-well ultra-low attachment plates (Corning #3473) for analysis for spheroid growth. For migration analysis these spheroids were transferred to regular adhesive 24-well culture plates. The size of the spheroids was measured every 3-days over a 15-day period. A SONY DSC-HX1 camera was attached to the microscope and the pictures were always taken with the same resolution and configuration: 2048x1536px, horizontal and vertical resolution of 72 dpi, Bit depth 24, Exposure time 1/30 s, Focal length 5 mm. The software IMAGE-J was used for the measurement of the diameters used for the calculations of area and volume.

### Synergy calculation and statistics

Statistical analysis was done using one-way ANOVA to compare different groups. Two-way repeated measures ANOVA was used for the experiments with several time points. Bonferroni post hoc test was utilized to compare differences between groups. The value of p was adjusted to the number of groups. Differences between two sets of data were considered statistically significant at *p* < 0.05 (95% CI). The combination index (CI) for n amount of treatments that were combined, was calculated using a adapted formula from Chou and Talalay [23, 24] which was used on normalized growth data, where v is cell viability in %.

$$ CI\left[ ndrugs\right]=\frac{\left[\sum \left[\frac{1}{V n}\right]\right]-\left[\frac{n-1}{100}\right]}{\left[\frac{1}{V1.. n}\right]} $$

## Results

*MK2206 does not lead to temozolomide/radiation sensitization in glioma monolayer cultures.*

**Fig. 1:**
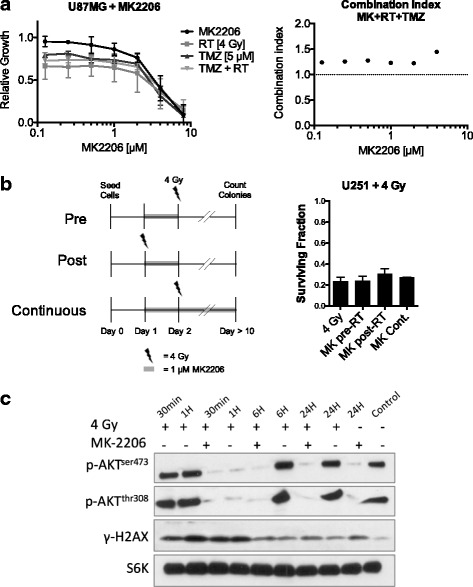
MK2206 does not lead to temozolomide/radiation sensitization in glioma monolayer cultures. **a** U87MG cells were treated for 72 h with MK2206 at indicated concentrations and treated with 5 μM TMZ or 4 Gy irradiation or the combination of both. The combination index of the triple combination was calculated at each time point. A CI < 0.8 indicates synergy, 0.8 < > 1.2 additive, > 1.2 antagonism. Data points represent means, ±SD (*n* = 3). **b** U251 cells were treated with 1uM MK2206 and or 4 Gy irradiation in different schedules. *Top*: MK was given 24 h before (Pre-RT) irradiation and plated for colony formation; *Middle*: cells were treated with MK and immediately irradiated (Post-RT), cells were plated 24 h later for colony formation; *Bottom*: cells were treated with MK 24 h before irradiation and cells were plated for colony formation in the presence of 1 μM MK2206. Columns represent means, ±SD (*n* = 2). **c** U87MG cells were treated with 1 μM MK and/or with 4 Gy irradiation and lysed at indicated time points

We investigated the efficacy of MK2206 at attenuating U87 glioma cell proliferation and found it to be effective at 1 μM and higher (Fig. 1a). Protein analysis showed complete AKT dephosphorylation at 1 μM (Fig. 1c). In the combination of MK2206 with 4 Gy irradiation or 5 μM TMZ no synergistic interaction was found. Synergy calculations show a combination index (CI) > 1.2, indicating a slight antagonism (Fig. 1a). Next we tested the combination with irradiation on clonogenic cell survival using U251 glioma cells. None of the different MK2206 treatment schedules showed a reduction in clonogenic survival (Fig. 1b). In line with these findings, the expression of γH2A.X phosphorylation was not increased at 24 h after 4 Gy irradiation in cells treated with MK2206 (Fig. 1c). The data show that in monolayer growing cell cultures MK2206 was not able to induce sensitization to DNA damaging therapies and actually showed antagonistic tendencies.

### Low dose MK2206 sensitizes long-term U87 multicellular spheroid cultures to irradiation and temozolomide

**Fig. 2:**
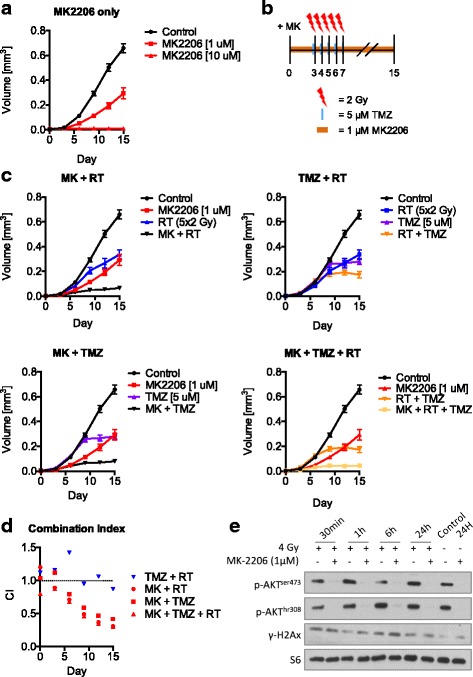
Low dose MK2206 sensitizes long-term U87MG multi-cellular spheroid cultures to irradiation and temozolomide. **a** U87 multicellular spheroids were treated for 15 days with 1 μM or 10 μM MK2206. Data points represent means, ±SD (*n* = 8 spheroids). **b**-**c** U87 spheroids were treated for 15 days with 1 μM MK2206 together with fractions of 5 μM TMZ or 2 Gy irradiation. Points are means, ±SD (*n* = 8 spheroids). **d** Combination index for all combinations of MK/TMZ/RT for each time point. CI < 1 indicates synergy, CI > 1 indicates antagonism. **e** U87 spheroids were treated with 1 μM MK and/or with 4 Gy irradiation and lysed at indicated time points

To further study the ability of MK2206 to sensitize glioma cells, we used the capability of U87 to easily form spheroids in low-attachment plates [25]. The growth of spheroids was inhibited after single exposure to 1 μM MK2206 and their growth was completely abrogated at 10 μM (Fig. 2a). Next, we treated the spheroids with fractionated irradiation in the presence of 5 μM TMZ and/or 1 μM MK2206. Spheroids were irradiated 3 days after start of MK2206 treatment, and TMZ was given 1 h prior to irradiation (Fig. 2b). MK2206 sensitized U87 spheroids to radiation with the lowest CI at 0.33 and 0.42 for TMZ. Furthermore, combining all three treatment modalities resulted in a strong synergy (CI > 0.33, Fig. 2c & d) which is dependent on MK, since TMZ and RT do not show any synergy (Fig. 2c). Spheroids showed complete pAKT inhibition and increased γH2A.X expression after irradiation combined with MK2206 (Fig. 2e). The data show that low-dose MK2206 can sensitize glioma spheroids to both irradiation and TMZ.

### High dose MK2206 inhibits glioma migration and invasion.

**Fig. 3:**
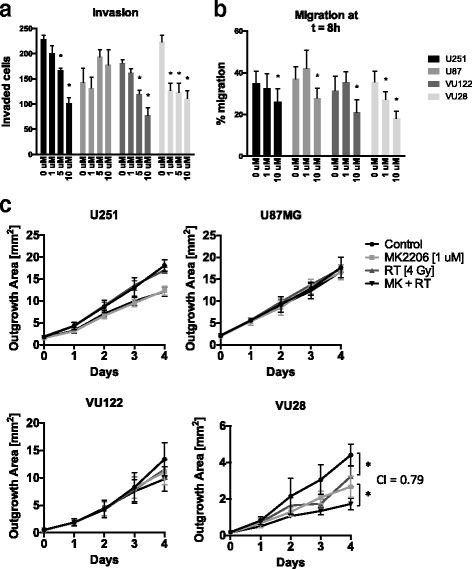
High dose MK2206 inhibits glioma migration and invasion. **a** Number of cells invaded through matrigel after 16 h in the presence of 1 μM, 5 μM, or 10 μM MK2206. Columns represent means, ±SD (*n* = 3), * = *p* < 0.05. **b** Percentage of scratch area remaining compared to 0 h. Columns represent means, ±SD (*n* = 10 replicates), * = *p* < 0.05. **c** Area of spheroid outgrowth. Spheroids were treated with 1 μM MK and 4 Gy irradiation and plated in regular culture plates. Data points represent means, ±SD, (*n* = 8 replicates), * = *p* < 0.05

An important aspect of glioma therapy resistance is the ability for the cells to invade and migrate throughout the brain. Therefore, we investigated the effect of AKT inhibition on the mobility of glioma cells on the established cell lines U87 and U251 and on the primary cell lines VU28 and VU122. In Fig. 3a the invasive capabilities of each cell line were quantified in the presence of increasing doses of MK2206. U251 and VU122 showed a decrease in invasion at a relatively high dose of 5 μM with a further decrease at 10 μM (*p* < 0.01). The invasion of VU28 was modestly attenuated at the low dose of 1 μM. Higher doses did not decrease the invasion further. Interestingly no inhibition of invasion was observed in U87 cells. Combining MK2206 (1 μM & 10 μM) with irradiation yielded an additive interaction for invasion inhibition for all cell lines (CI > 0.8) (Additional file 1: Figure S1A). Wound healing experiments were performed to assess migration inhibition, using 1 μM and 10 μM MK2206 at multiple time-points up to 8 h after start treatment (Additional file 1: Figure S1B). All cells showed a modest yet significant inhibition of migration at 10 μM (Fig. 3b). VU28 was the only cell line to have inhibition of migration at a low dose of 1 μM. Furthermore, we set up a spheroid outgrowth assay to study the effect of (low dose) AKT inhibition combined with irradiation on a longer time-scale of 4 days (Additional file 1: Figure S1B). Figure 3c shows that the VU28 cell line is the only cell line in which both radiation and MK alone significantly reduced migration and when combined leads to a mild synergistic interaction (CI = 0.79). These results show that AKT inhibition and irradiation do not preferentially synergize in glioma mobility inhibition. However, strong attenuation of the AKT pathway does lead to a decrease in invasion and a mild decrease in migration.

## Discussion

In the present study, we investigated the effect of AKT inhibition by MK2206 on adherent growing human glioma cells and on multicellular spheroids. The most important finding is that low dose MK2206 is able to synergistically sensitize glioma spheroids to the current standard treatment modalities in GBM therapy, i.e. irradiation and TMZ. This is in contrast to adherent growing which showed at best modest additivity for the combinatorial treatments. This has not described earlier in the context of radiosensitization since the golden standard for radiosensitization has always been monolayer clonogenic assays. Hence, studies for evaluation of radiosensitizing agents should take in to account the biological and physiological limitations of monolayer cultures. Cells growing in multicellular organoid structures more faithfully resemble the context of real life tumors. Cells in this context deal with complex interactions due to heterotypic cell-to-cell contact, signaling, extracellular matrix deposition and intracellular structure. This micro milieu results in gradients of oxygen, nutrients and biomolecules can result in hypoxia/oxidative stress [26, 27], which has been shown to culminate to an increase of the autophagic flux near the core of spheroids [28]. Cheng and colleagues have previously shown that the treatment of glioma cells with MK2206 preferentially leads to an increased autophagic flux and that this could be increased synergistically with gefitinib leading to autophagic cell death [29, 30]. In our study RT and TMZ were used to induce genotoxic stress (DNA-damage) which is a well-known inducer of autophagy [31, 32]. We therefore hypothesize that AKT inhibition together with RT/TMZ in spheroids tips the autophagic equilibrium towards autophagic cell death which cannot be achieved in adherent cells. The presence of these mechanisms and the concomitant different phenotype makes spheroids a preferred model over adherent monolayer growing cells for studying radiosensitizing potential of targeted agents [33]. Recently, a study has shown similar results where the authors show that AKT inhibition does not radiosensitize U87 monolayer cells but does sensitize primary glioma stem-like cell cultures to irradiation [34]. U87 cells grown as spheroids have been shown to have elevated levels of the stem cell marker CD133 [35], indicating another mechanism of how the structural organization of cells influence internal signaling and drug response.

The ability of glioma cells to invade into the brain and migrate to different regions of the brain is one of the most important physical means of therapy resistance. AKT is thought to stimulate cell migration through signaling routes [36]. Cellular invasiveness can be inhibited by irradiation, which has been reported before [37]. However, our data shows a modest attenuation of glioma cell mobility through AKT inhibition and shows synergy in only one of the four cell lines tested (Figure 3). Nevertheless, this modest inhibition together with the synergistic effect the combination therapy shows on growth inhibition warrant further investigation in an orthotopic in vivo glioma model. Cheng and colleagues have shown MK2206 alone to have inhibitory effects on in vivo glioma growth [30].

## Conclusions

Taken together, low dose MK2206 enhanced the effect of radiotherapy and TMZ on brain tumor spheroids in vitro which was not seen in adherent growing cell lines. Furthermore, High dose MK2206 inhibited migration and invasion of glioma cells and also synergized with irradiation in a primary GBM line. Our findings indicate the AKT pathway to be a promising target to be combined with the current standard of care for GBM therapy.

## Additional file

Additional file 1: Figure S1. A) Number of cells invaded through matrigel after 16 h in the presence of 4 Gy combined with 1 μM (Left) or 10 μM (Right) MK2206. Error bars represent SD over 3 replicates, * = *p* < 0.05. B) U87 cells untreated. Migration of U87 cells into wound up to 8 h (Left). Migration of U87 cells out of attached spheroids up to 4 days after spheroid plating (Right). (PDF 356 kb)

## Abbreviations

GBM: Glioblastoma
RT: Radiotherapy
TMZ: Temozolomide
MGMT: O6-methylguanine–DNA methyltransferase
PI3K: phosphatidylinositide 3-kinase
DDR: DNA-damage response
NHEJ: Non-Homologous end-joining
CI: Combination Index
