# Supplementary figures and images for: The allosteric AKT inhibitor MK2206 shows a synergistic interaction with chemotherapy and radiotherapy in glioblastoma spheroid cultures
Source: BMC Cancer. 2017 Mar 21;17:204. doi: 10.1186/s12885-017-3193-9 (PMC5359921; doi:10.1186/s12885-017-3193-9)

A. Invasion MK+RT

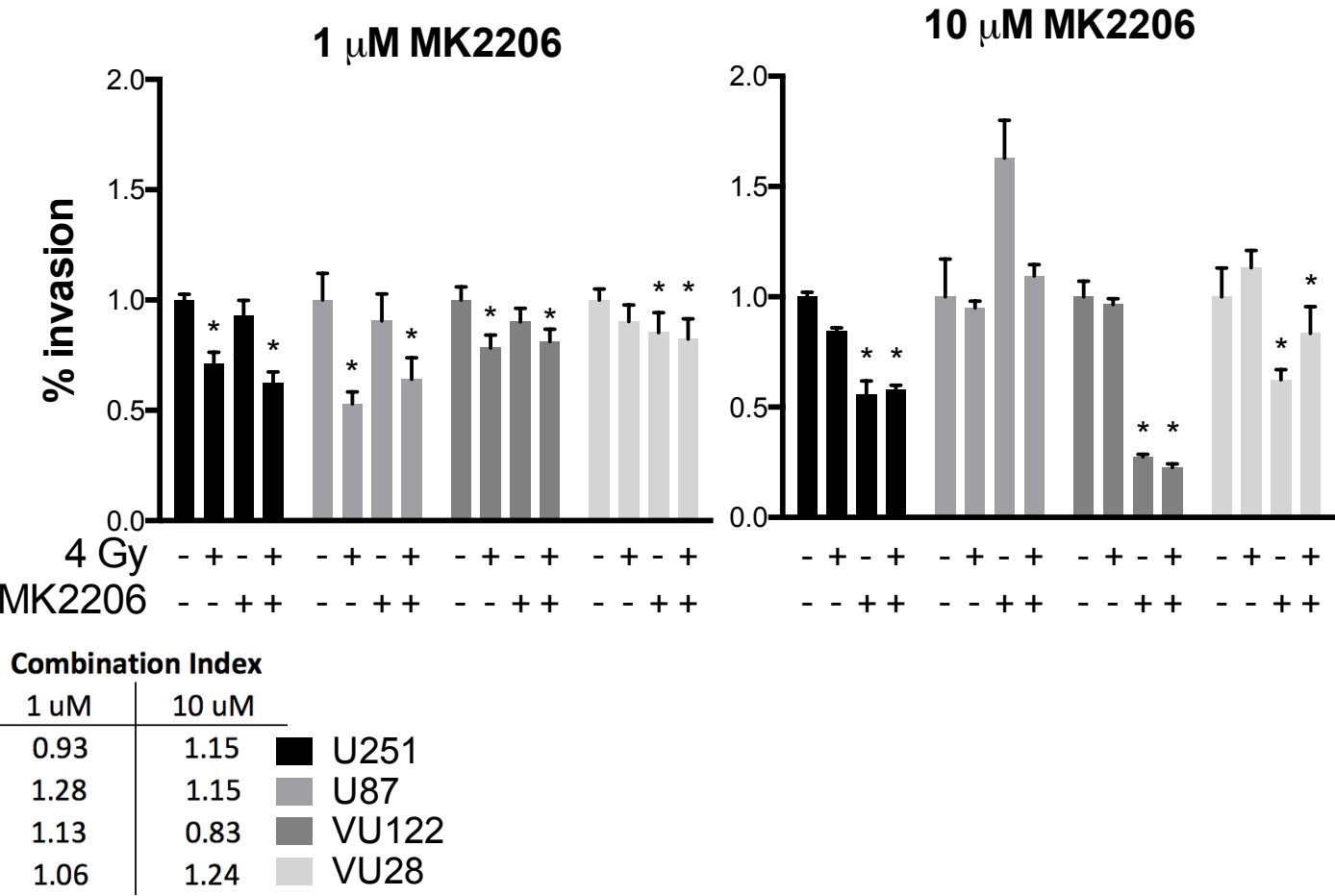

B. Migration (wound healing (Left) spheroid outgrowth (right) U87 cells

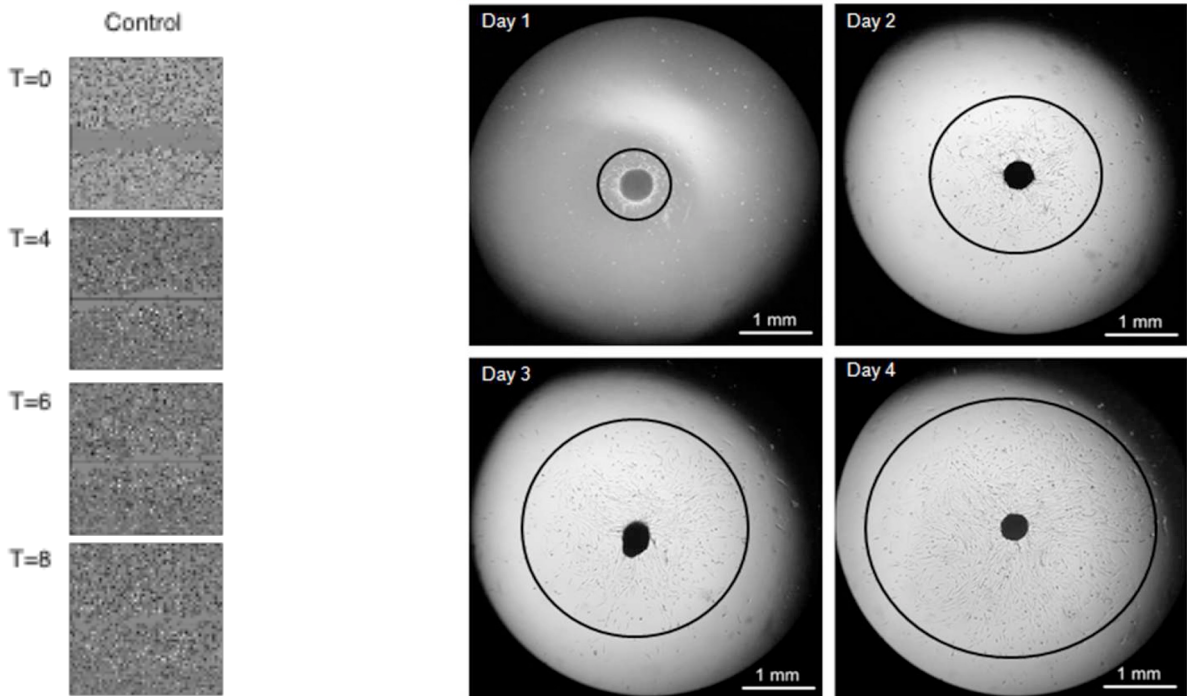

Supplement: Additional file 1: Figure S1. — A) Number of cells invaded through matrigel after 16 h in the presence of 4 Gy combined with 1 μM (Left) or 10 μM (Right) MK2206. Error bars represent SD over 3 replicates, * = p < 0.05. B) U87 cells untreated. Migration of U87 cells into wound up to 8 h (Left). Migration of U87 cells out of attached spheroids up to 4 days after spheroid plating (Right). (PDF 356 kb) [file 12885_2017_3193_MOESM1_ESM.pdf]
